# Analysis of Benzene-Induced Effects on *Rhodococcus* sp. 33 Reveals that Constitutive Processes Play a Major Role in Conferring Tolerance

**DOI:** 10.1100/tsw.2009.29

**Published:** 2009-03-31

**Authors:** Tony Gutiérrez, Robert Learmonth, Iain Couperwhite

**Affiliations:** ^1^ School of Biotechnology and Biomolecular Sciences, The University of New South Wales, Sydney, NSW 2052, Australia; ^2^ Department of Biological and Physical Sciences Center for Systems Biology, University of Southern Queensland, Toowoomba, QLD 4350, Australia

**Keywords:** Rhodococcus, benzene, solvent tolerance, cell membrane fluidity, extracellular polysaccharide

## Abstract

Most studies investigating mechanisms that confer microorganisms with tolerance to solvents have often focused on adaptive responses following exposure, while less attention has been given to inherent, or constitutive, processes that prevail at the onset of exposure to a toxic solvent. In this study, we investigated several properties of the highly solvent-tolerant bacterium *Rhodococcus* sp. 33 that confer it with a tolerance to high concentrations of benzene. When challenged with liquid benzene, the growth of both nonadapted and adapted cells was decreased by 0.25¨C0.30% (v/v) liquid benzene, and higher concentrations (≤0.35% v/v) produced a complete cessation in the growth of only nonadapted cells. When exposed to presolubilized benzene, nonadapted cells tolerated ≥1000 mg/l, whereas adapted cells tolerated >1400 mg/l. Measuring the cell membrane fluidity of the cells during these exposure experiments showed that at the onset of exposure, the membranes of adapted cells were less affected by benzene compared to nonadapted cells, although these effects were insignificant in the long term. Several benzene-sensitive mutants were generated from this *Rhodococcus*, two of which were unable to degrade benzene, yet they still tolerated 500¨C800 mg/l. This confirmed our earlier work suggesting that the benzene-degradation pathway of this organism plays a minor role in tolerance. Under the phase and transmission electron microscope, the mutants were found to have lost the ability to produce extracellular polymers, and many cells appeared pleomorphic, containing intracellular membrane invaginations and mesosome-like structures. As will be discussed, these results identify important functions of the cell membrane, the cell wall, and extracellular polymer in their native state (i.e., before exposure) in conferring this organism with tolerance to benzene.

### INTRODUCTION

Numerous advances have been made in recent years in our efforts to enhance the bioremediation of pollutants at contaminated sites. However, an important problem still encountered is the inherent toxicity of some pollutants to the microorganisms responsible for their degradation. This is especially true of aromatic hydrocarbons, such as benzene, toluene, and the xylene isomers (BTX). Even at low concentrations, these compounds can cause total inhibition of microbial activity or lysis of cells[1]. In order for degradation to occur, however, the microorganisms must tolerate exposure to these compounds. Hence, much research has been devoted to understanding the interactions between organic solvents and microbial cells, with a view to improving the tolerance and degradative ability of microorganisms[2].

Lipophilic compounds, like BTX, tend to partition within the lipid bilayer of microorganisms, thereby causing an overall increase in cell membrane fluidity and often leading to the functional impairment of the membrane and possibly cell death[3,4]. Some microorganisms, however, are able to tolerate and resist membrane damage when exposed to toxic solvents, even at high concentrations[5]. This is accomplished by inducing an increase in the ratio of saturated/unsaturated fatty acids, *trans/cis* unsaturated fatty acids, and/or protein/lipid within their cell membranes[3,4,6]. These adaptive responses tend to increase the rigidity of cell membranes, thereby countering the fluidizing effect of organic solvents and, in turn, reducing their partitioning within the lipid bilayer[4]. Adaptive responses, however, take time. Whether it is seconds, minutes, or hours, adaptation following immediate exposure to a toxic solvent is preceded with a period that places the organism in a vulnerable state. Since the cell membrane and cell wall, if present, are the sites where a solvent will come into first contact with a cell, these subcellular layers are expected to play a crucial role in conferring tolerance prior to the onset of adaptation if the organism survives.

In recent years, the rhodococci have been recognized for their robust nature and ability to survive exposure to a variety of environmental insults. Various species have been isolated and identified with an ability to tolerate and, like the pseudomonads, degrade various organic pollutants[7,8]. More recently, some species of *Rhodococcus* have been isolated, exhibiting very high levels of tolerance to toxic solvents, e.g., benzene[9,10,11]. *Rhodococcus* sp. 33, used in the present study, is able to tolerate and grow on benzene at supersaturating concentrations (>1800 mg/l)[12]. Here, we conducted a comparative evaluation of the physiological and morphological characteristics of *Rhodococcus* sp. 33 and its benzene-sensitive mutants in order to identify the inherent and adaptive mechanisms that this bacterium employs for tolerating and adapting to high concentrations of benzene.

### MATERIALS AND METHODS

#### Organism and Growth Conditions

The organism used in this study was *Rhodococcus* sp. 33, a bacterium that was previously isolated from a contaminated site in Sydney, Australia, for its ability to degrade benzene[13]. The organism was stored at –80°C in 30% glycerol, from which streaked plates were prepared on nutrient agar (NA). Inocula were prepared by transferring a colony of the cells into a 250-ml conical flask containing 50 ml of a sterile phosphate and ammonium salts (PAS) liquid medium, as previously described[13]. This is a chemically defined medium containing the following: 16 ml 1 *M* $NH_4Cl$, 10 ml 1 *M* $K_2HPO_4$, 5 ml 1 *M* $KH_2PO_4$, and 365 ml of Milli-Q grade water buffered to pH 6.8. Prior to inoculation, 4 ml of a filter-sterilized (0.2 µm) salts solution was added per 400 ml of PAS. The stock salt solution was prepared by dissolving 37.5 g $MgSO_4.7H_2O$, 5.0 g $MnSO_4.H_2O$, 5.0 g $FeSO_4.7H_2O$, 0.3 g $CaCl_2.2H_2O$, and 1.0 g ascorbic acid per liter of Milli-Q grade water. Inocula (5% v/v) were transferred into fresh PAS flasks and incubated with shaking (100 rpm) at 28°C. Unless otherwise indicated, all culture flasks were plugged with nonabsorbent cotton wool to allow for the diffusion of air. The carbon sources used to grow the organism were 1% (w/v) mannitol (Man) and/or benzene (Man+Benz or Benz) at various concentrations. For the isolation of plasma membrane fractions, glycine was added to the medium prior to autoclaving (see below). When benzene was supplied via the vapor phase, a test tube was placed inside the PAS flask, to which benzene was added to allow the vapors to diffuse freely into the medium and headspace.

### Generation and Enriched Selection of Benzene-Sensitive Mutants

Benzene-sensitive mutants were generated from the *wild-type* (*w-t*) strain (i.e., *Rhodococcus* sp. 33) using ethyl methanosulfonate (EMS), as adapted from the method by Eisenstadt et al.[14]. This involved subjecting the cells to a 4% solution of freshly prepared EMS (Sigma), prewarmed to 37°C, for 2 h. The cells were then washed twice with 0.1 *M* phosphate buffer. To select for benzene-sensitive mutants (i.e., nongrowing cells), the cells were resuspended in a 50-ml volume of LB (Luria-Bertani) broth supplemented with 200 mg/l of presolubilized benzene and 2 mg/l of ampicillin. The benzene was presolubilized into the medium for approximately 2–3 h with shaking (100 rpm) prior to inoculating. After 4 h of incubation (28°C, 190 rpm), the cells were harvested and appropriate dilutions performed for replica spread plating on LB agar medium. Plates were incubated at room temperature in a cotton wool–plugged desiccator for 72 h with benzene supplied via the vapor phase. For comparative selection of sensitivity to benzene, duplicates of these plates were maintained under identical conditions without benzene. Benzene-sensitive mutants were selected as colonies of less than 0.5 mm in diameter from replicate plates incubated without benzene.

### Effect of Benzene on Growth Rate and Membrane Fluidity

Cells grown to the mid-log phase in Man medium (i.e., benzene nonadapted cells) were harvested and washed twice with 0.1 *M* potassium phosphate buffer (pH 7). Equal aliquots of the washed cells were transferred into a series of 250-ml Teflon centrifuge tubes containing 100 ml of Man medium and increasing concentrations of presolubilized benzene — 0, 200, 300, 500, 800, 1000, and 1200 mg/l. Prior to addition of the cells, benzene was presolubilized into the medium as described above. The tubes were hermetically sealed and incubated with shaking at 28°C for 8 h. Samples for viable count measurements and analysis of membrane fluidity (see below) were taken at the time of inoculation and periodically thereafter. For comparison, the experiment was repeated using cells that had been grown on benzene (~200 mg/l) as the sole carbon source when supplied via the vapor phase, i.e., benzene-adapted cells. All experiments were performed in triplicate.

To determine the effect of exposing growing cells to "shock" concentrations of benzene, cells grown in Man medium were prepared as above and inoculated into eight 250-ml Erlenmeyer flasks containing 50 ml of Man medium. The flasks were plugged with nonabsorbent cotton wool and the cultures allowed to grow with shaking (150 rpm) at 28°C to a cell turbidity of 0.20–0.35 (OD at 600 nm; tenfold dilution). A volume of liquid benzene — 0, 0.05, 0.08, 0.10, 0.15, 0.20, 0.25, 0.30, and 0.35% (v/v) final concentration — was then directly added into the growing cultures. Growth of the cultures was monitored ($OD_{600}$) throughout the 29-h duration of each experiment to observe any effects by benzene on growth rate. Samples were also examined by phase contrast, light, and transmission electron microscopy. The experiment was repeated, this time using benzene-adapted cells that had been grown on ~200 mg/l benzene when supplied via the vapor phase. All experiments were performed in triplicate.

The ability of each mutant strain to grow on benzene as a sole carbon source was evaluated in PAS medium. For this, cells grown to the mid-log phase in Man medium were harvested and washed twice before inoculating into 250-ml Erlenmeyer flasks containing 50 ml of PAS. Benzene was supplied via the vapor phase, as described above. Growth was monitored ($OD_{600}$) over several days.

### Microscopy

Samples for viewing under the transmission electron microscope (TEM) with negative staining were prepared by suspending the samples in distilled water and then mixing with an equal volume of sodium phosphotungstic acid (PTA) for 10 sec. The stained suspensions were mounted on a 400-mesh copper grid coated with collodion/carbon. After approximately 30 sec, the grid was touch dried with filter paper and allowed to air dry (~15 min) before viewing using a Hitachi H-7000 TEM.

Samples for thin-sectioning TEM were fixed with a solution of 2.5% glutaraldehyde in 0.1 *M* sodium cacodylate buffer for 1 h at 4°C. The samples were then washed three times with cacodylate buffer and once with distilled water. For the embedding process, an equal volume of the pellet sample was mixed with a 2% agar solution on a microscope slide. Once solidified, 1-mm cubed blocks were cut from the sample agar matrix and placed into a small volume of 2% osmium tetroxide ($OsO_4$) fixative for 2 h at 4°C. The blocks were then rinsed in a solution of 2% aqueous sodium acetate for 30 min, then postfixed in a 2% uranyl acetate solution for 1 h. To observe for the expression of surface-expressed polysaccharide, ruthenium red (1% w/v) was initially applied to samples and incubated in the dark for 2 h at 4°C. Ruthenium red (0.1% w/v) was again added to the samples during their treatment with 2% $OsO_4$ for 3 h at 21°C in the dark. Sample blocks stained with or without ruthenium red were then dehydrated in a series of ethanol solutions and acetone as follows: 50% ethanol + 0.1% NaCl (15 min), 70% ethanol (15 min), 95% ethanol (15 min), 100% ethanol (15 min), 100% ethanol (30 min), and 100% acetone (1.5 h). After dehydration, the blocks were then treated with a Spurr's epoxy resin for resin infiltration, further dehydration, and finally embedding. The epoxy resin contained the following: 23.6% vinylcyclohexene dioxide, 14.2% diglycidyl ether of polypropylene glycol, 61.3% nonenyl succinic anhydride, and 0.9% dimethylamine ethanol. The sample blocks were then treated to a 1:1 acetone/resin mixture for 6 h, then 9:1 for 12 h, and 100% resin for 6 h at 4°C. Individual sample blocks were placed into polythene capsules with freshly prepared resin and allowed to cure for 48 h in a 60°C oven. Gold sections (90–120 nm thick) were cut from embedded samples using glass knives and a Reichert-Jung Ultracut microtome. Sections were mounted onto 400-mesh copper grids, coated with collodion/carbon, and then stained with lead citrate and uranyl acetate. Examination of the sectioned material was performed using a Hitachi H-7000 TEM.

### Membrane Fluidity Analysis

Membrane fluidity was assessed by measurement of generalized polarization (GP) using the fluorescent membrane probe laurdan (6-dodecanoyl-2-dimethylaminonaphthalene), as developed for application with microorganisms[15,16]. When necessary, cell cultures were diluted to $OD_{600}$ = 0.3 (0.1 dilution) for fluorescent probe labeling. Five microliters of laurdan (Molecular Probes, Eugene, OR), from a 1-m*M* stock solution in ethanol, was added to 5 ml of cell culture. This was then incubated for 30 min at room temperature in the dark prior to spectrofluorometric analysis.

## RESULTS

### Isolation of Benzene-Sensitive Mutants

The viable count before and after mutagenesis was $5.3 \times 10^9$ and $1.5 \times 10^9$ cells/ml, respectively, resulting in a cell kill of 72%. Using our method for generating benzene-sensitive mutants from *Rhodococcus* sp. 33, the mutation frequency was calculated to be $2.7 \times 10^{-3}$.

To select for benzene-sensitive mutants, the addition of ampicillin (2 mg/ml) during exposure of the EMS-treated cells with benzene resulted in a 33% reduction in the viable cell count. From the remaining population of cells, i.e., the nongrowing benzene-sensitive population, a total of 48 colonies were isolated as benzene sensitive, based on their small colony size of ≤0.5 mm. Subsequent experiments to assert their stability as benzene sensitive showed that only four of the 48 isolates maintained this characteristic. Three of these mutants displayed the highest benzene sensitivity, as they did not develop colonies when exposed to vapor concentrations of benzene. These mutant strains were labeled M1, M2b, and M5b. One mutant developed colonies of ~0.5 mm in diameter and was labeled M2c. Growth of these mutants in the absence of benzene resulted in colony sizes similar to that of the *w-t*. Another two mutant strains, labeled M3 and M6b, were also included in our selection because, although they developed colonies similar in size to the *w-t* when exposed to benzene, they displayed a rough colony morphology that at the time suggested a loss in their ability to produce extracellular polymer.

### Tolerance to Presolubilized and "Shock" Concentrations of Benzene

The tolerance of nonadapted cells of the *w-t* and mutant strains was examined by measuring their growth ($OD_{600}$) during 8-h exposure to increasing concentrations of presolubilized benzene in PAS medium. As shown in Fig. 1, none of the mutants tolerated 1200 mg/l benzene, the *w-t* (nonadapted) tolerated up to 1000 mg/l benzene, and all six mutant strains exhibited reduced tolerance limits — M3 and M6b (≤800 mg/l), M2c (≤700 mg/l), M1 and M5b (≤500 mg/l), M2b (≤400 mg/l).

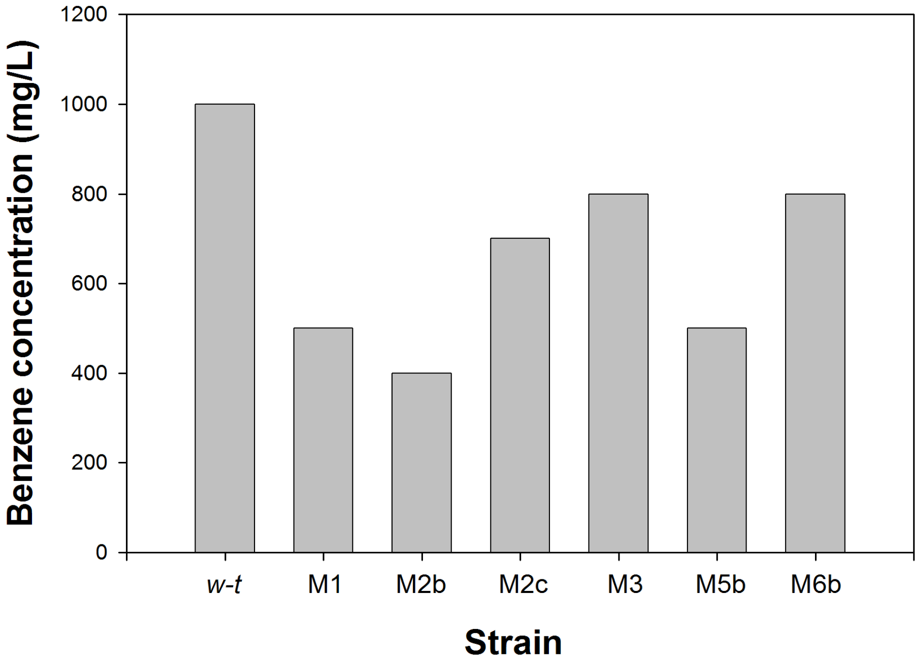

**FIGURE 1.** Tolerance limits of the *w-t* and its benzene-sensitive mutants to benzene.

We also examined the effect of exposing each mutant strain to a continuous supply of benzene (via the vapor phase) in PAS medium amended with mannitol (Man+Benz medium). Compared to growth without benzene in Man medium (Fig. 2A), all of the strains, except M2b and M5b, displayed similar growth rates in Man+Benz medium (Fig. 2B). The presence of benzene, at initial concentrations of 190 mg/l, produced a long lag phase (~45 h) and a markedly slower growth rate in cells of strain M5b. With M2b, it caused a slight decrease to the growth rate of the cells. Interestingly, when cell suspensions of M6b were allowed to stand unshaken, the cells settled out of solution within minutes. Although not shown, we observed a similar

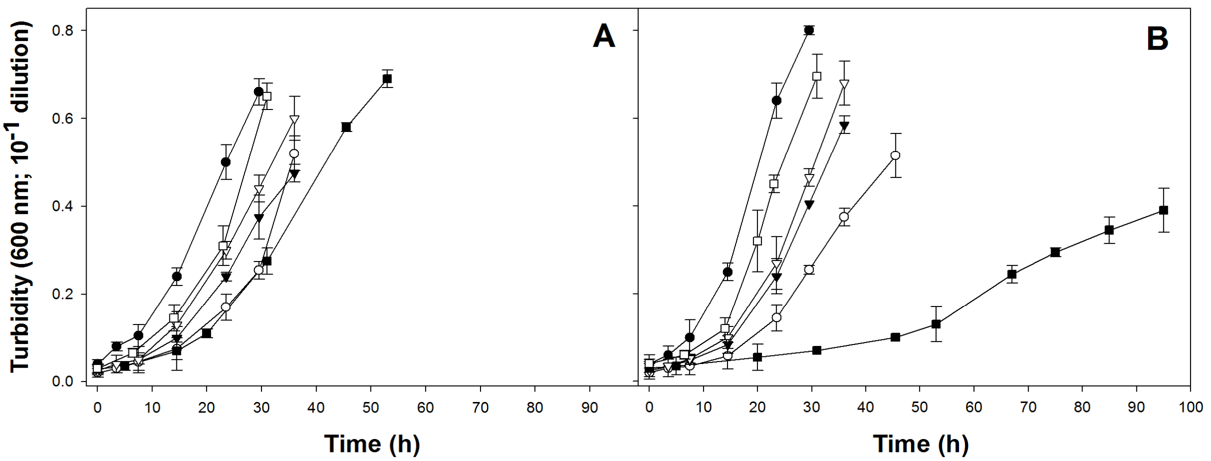

**FIGURE 2.** Growth of each mutant strain on mannitol (A) and Man+Benz (B) medium. M1 (●), M2b (○), M2c (▼), M3 (▽), M5b (■), and M6b (□). Where SD error bars are not shown, it is because they were smaller than the symbol.

effect in cells of the other five mutant strains, which settle out of solution only after standing for several hours. Comparatively, cells of the *w-t* remained in suspension when left standing for several days. As will be discussed below, this differential flocculation behavior between the *w-t* and mutant strains may reflect the expression of extracellular polymer.

The effect of challenging nonadapted and adapted cells of the *w-t* to "shock" additions of liquid benzene was investigated in PAS medium. As shown in Fig. 3A, "shock" additions of up to 0.20% (v/v) benzene had little effect on Man-grown (nonadapted) cells. Additions of 0.25–0.30% (v/v) benzene, however, produced an immediate cessation in growth that subsequently resumed after approximately 3 h. The addition of 0.35% (v/v) benzene resulted in cell death. On the other hand, benzene-adapted cells were unaffected when challenged with up to 0.20% (v/v) benzene (Fig. 3B). Additions of 0.25–0.35% (v/v) benzene produced a short (~2 h) interruption in cell growth followed by their return to near normal growth rates.

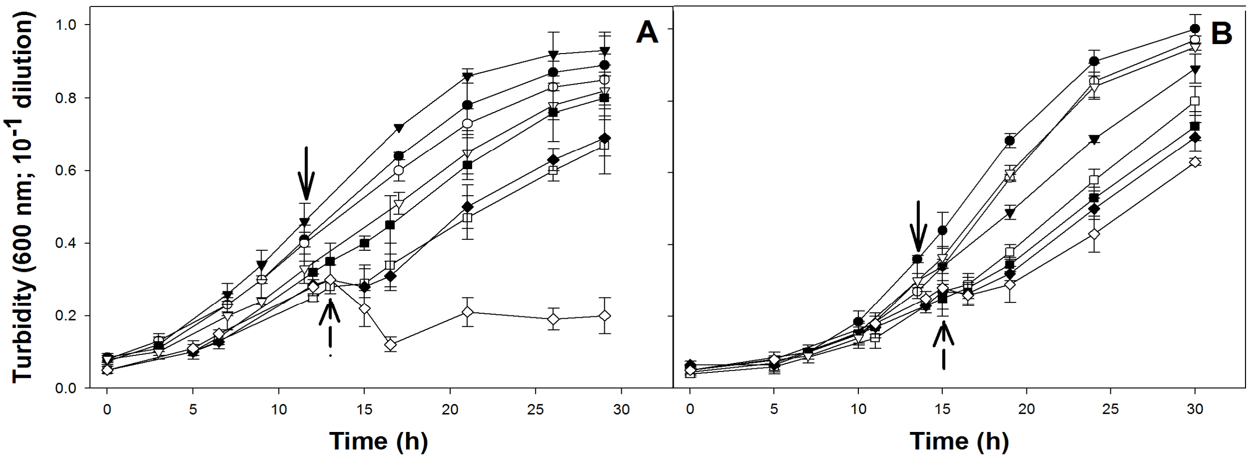

**FIGURE 3.** Cells of the *w-t* that had been grown on mannitol (A) and benzene (B) were challenged with increasing liquid concentrations of benzene — 0.05% (●), 0.08% (○), 0.10% (▼), 0.15% (▽), 0.20% (■), 0.25% (□), 0.30% (◆), and 0.35% (◇). The arrows denote the time when benzene was added (solid arrow for 0.05–0.15%; dotted arrow for 0.20–0.35%). Where SD error bars are not shown, it is because they were smaller than the symbol.

### Growth on Benzene as a Sole Carbon Source

The ability of each mutant strain to grow on benzene as a sole carbon source was evaluated in PAS medium amended with benzene (effectively 190 mg/l) as the sole carbon source. As shown in Fig. 4, strains M2b, M2c, and M6b grew well on benzene, whereas strains M1 and M3 did not. Strain M5b also grew on benzene, however, this was preceded by a very long lag phase lasting ~70 h.

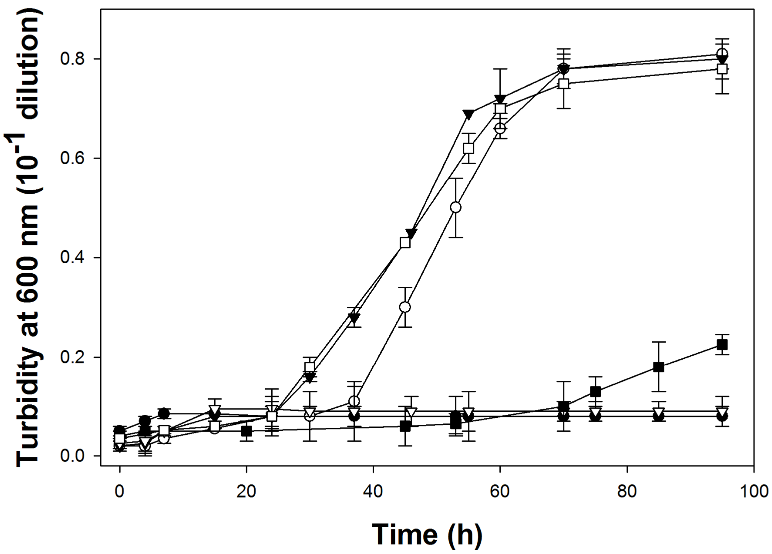

**FIGURE 4.** Growth of each mutant strain on benzene as the sole carbon source — M1 (●), M2b (○), M2c (▼), M3 (▽), M5b (■), M6b (□). Where SD error bars are not shown, it is because they were smaller than the symbol.

### Cell Morphology of the *w-t*

The cell morphology of the *w-t* was monitored by phase contrast microscopy after a suspension of the cells was layered alongside a drop of liquid benzene on a microscope slide. Within minutes, the cells became increasingly more transparent and "blebs" appeared to form at the terminal pole positions of some cells. As observed in Fig. 5, many cells had formed into well-developed protoplasts after 10-min exposure. This was most prominent for cells located within close proximity of the interfacial layer, i.e., the boundary between the aqueous (panel A) and nonaqueous (panel B) phases. Cells that remained opaque appeared to have assumed a pleomorphic morphology. No cells, however, were observed to have traversed the interfacial boundary and entered the nonaqueous (i.e., benzene) phase.

The cell morphology of the *w-t* was found to be different when it was grown with and without benzene. As shown in Fig. 6, cells grown with benzene displayed a coccoid morphology (A), whereas they appeared as thick rods when grown on mannitol (B).

Cells of the *w-t* grown in Man or Benz medium were found to express cell-bound extracellular polymer. The electron micrographs in Fig. 7 show the polymer as a thick layer surrounding the cells. The polymer fused into clumps and appeared in high contrast in ruthenium red–stained cells (Fig. 7A), seemingly an artifact caused by the staining technique. Micrographs of cells fixed and stained without employing ruthenium red showed the polymer as a thick hair-like layer (Fig. 7B).

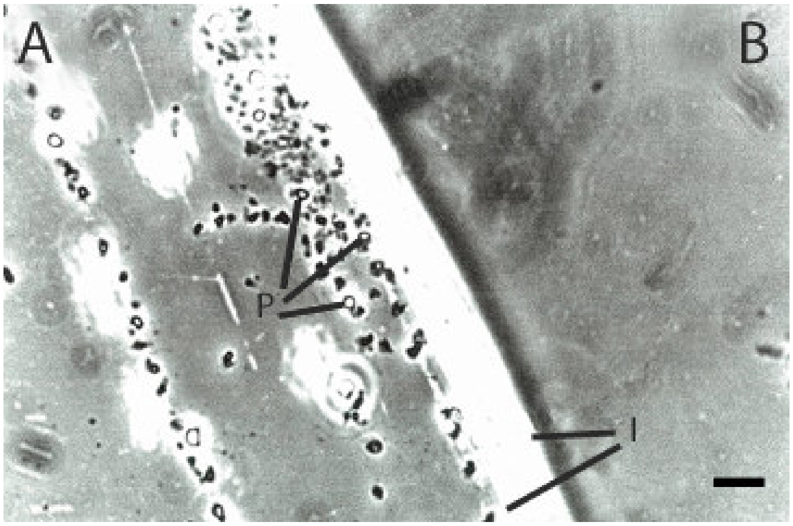

**FIGURE 5.** Phase contrast micrograph showing the interface (I) between a cell suspension of the *w-t* (A) and benzene (B). After only 10-min exposure to benzene, cells are shown to have formed into protoplasts (P) and appear in greater numbers nearest to the interfacial boundary. Bar = 5 µm.

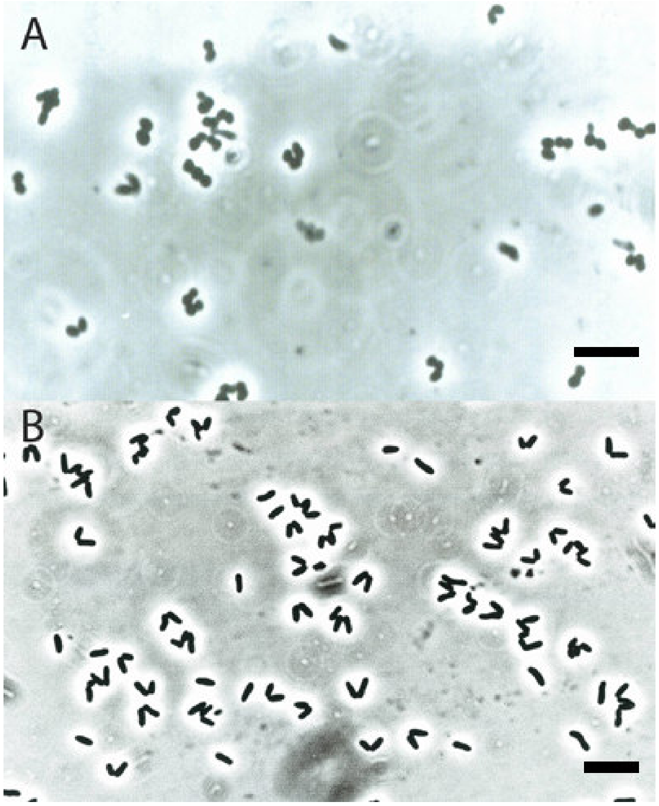

**FIGURE 6.** Phase contrast micrographs displaying the cell morphology of the *w-t* when the cells were grown on benzene (A, coccoid shape) or on mannitol (B, thick rods). Bar = 5 µm.

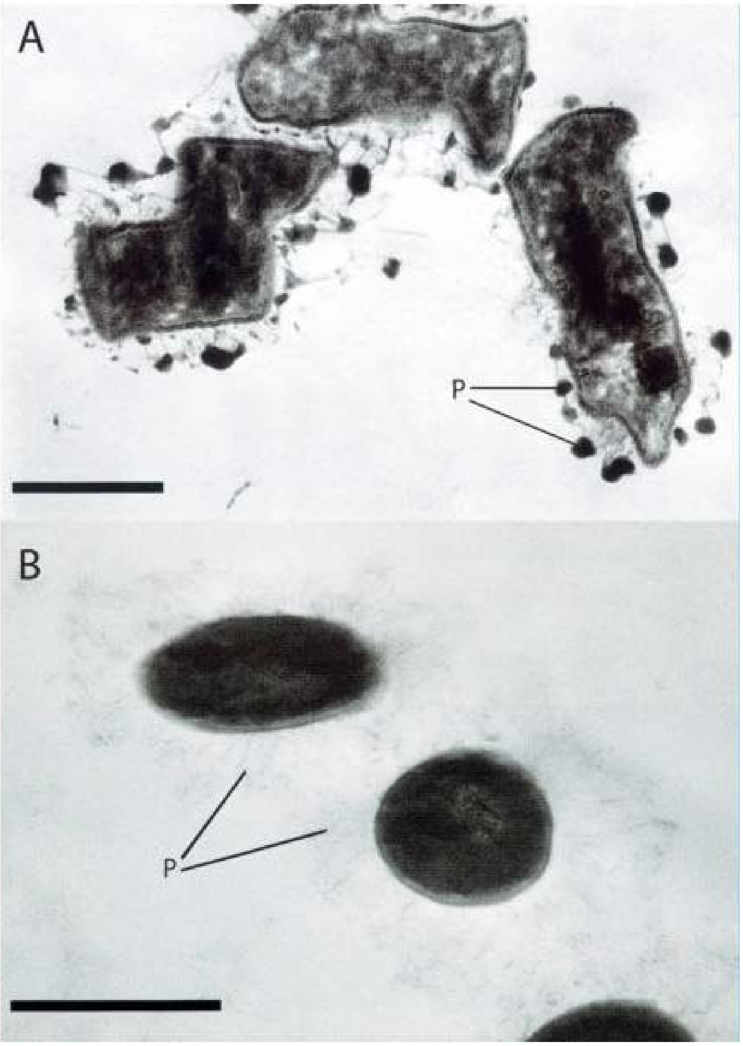

**FIGURE 7.** TEMs of thin-sectioned samples of the *w-t*. Cells stained with ruthenium red (A) appear pleomorphic and surrounded by a layer of clumped polymer (P). Cells stained with $OsO_4$ (B) appear to maintain their typical shape and are surrounded by a layer of polymer which, although it stained weakly, appear as a thick hair-like layer (P). Bar = 0.5 µm.

### Cell Morphology of the Mutants

Phase micrographs of each mutant grown in Man medium are shown in Fig. 8. Cells of M2b, M2c, M5b, and M6b displayed a pleomorphic morphology. M2b appeared as short thick rods, M2c and M6b as long thick rods, and M5b as long rods. Cells of M3 appeared as short thick rods, although not pleomorphic. Only cells of M1 appeared similar to the *w-t*. All of the mutants, except M1 and M2c, tended to produce clusters. This was particularly evident with M3 and M5b, and to a lesser extent with M2b and M6b. Interestingly, many of the cells that formed these clusters displayed a patchy translucence, i.e., the cells appeared striated. Cells of mutants M1, M2b, and M6b stained (as expected) positive with the Gram reaction, whereas mutants M2c, M3, and M5b stained variably (i.e., both positive and negative). As discussed below, these observations indicate possible differences in the expression of extracellular polymer causing different flocculation characteristics, as well as differences in cell wall thickness and integrity.

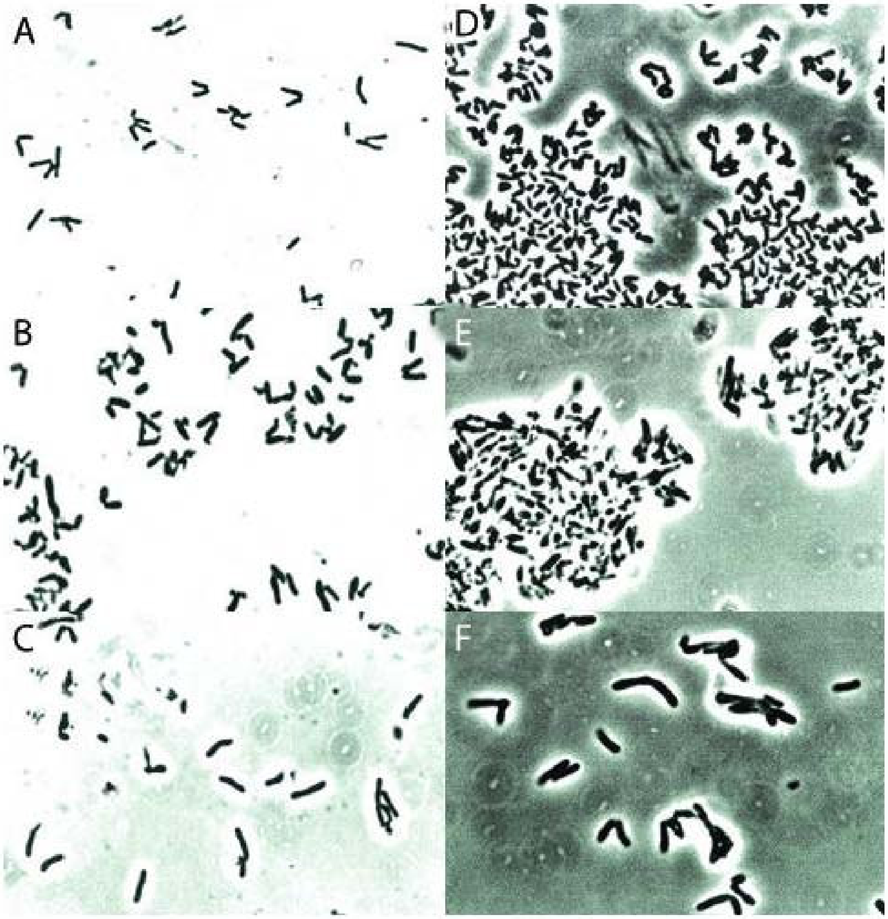

**FIGURE 8.** Phase contrast micrographs showing the cell morphology of each mutant strain — M1 (A), M2b (B), M2c (C), M3 (D), M5b (E), and M6b (F).

Fig. 9A shows a thin-sectioned TEM of an M5b cell illustrating the absence of extracellular polymer. Mutants M2c, M3, and M6b were also found to be devoid of polymer (not shown). The micrograph also shows the presence of intracellular invaginations and "mesosome-like" structures, both of which were frequently seen in all of the mutant strains when grown in either the presence or absence of benzene. These structures generally appeared variable in size, convoluted and elongated, and in some cells they were found localized within the nucleoid. Fig. 9B shows a thin-sectioned micrograph of M2b showing the cell undergoing asymmetric septation. Here also, extracellular polymer appears weakly expressed around the cell surface.

### Effects of Benzene on the Cell Membrane Fluidity of the *w-t*

Fig. 10 shows benzene effects on the GP value in membranes of nonadapted (A) and adapted (B) cells of the *w-t*. Compared to benzene-adapted cells, nonadapted cells showed a greater susceptibility to benzene, particularly when "shocked" with ≥600 mg/l. Within the first 3 h, the GP decreased, particularly in the 1000-mg/l treatment. Thereafter, the GP either stabilized or increased. With benzene-adapted cells, the GP value also decreased, although mainly within the first hour after exposure. Thereafter, the GP in these cells increased back to approximately initial values. Overall, the GP of nonadapted cells was more affected by "shock" exposures to benzene compared with benzene-adapted cells.

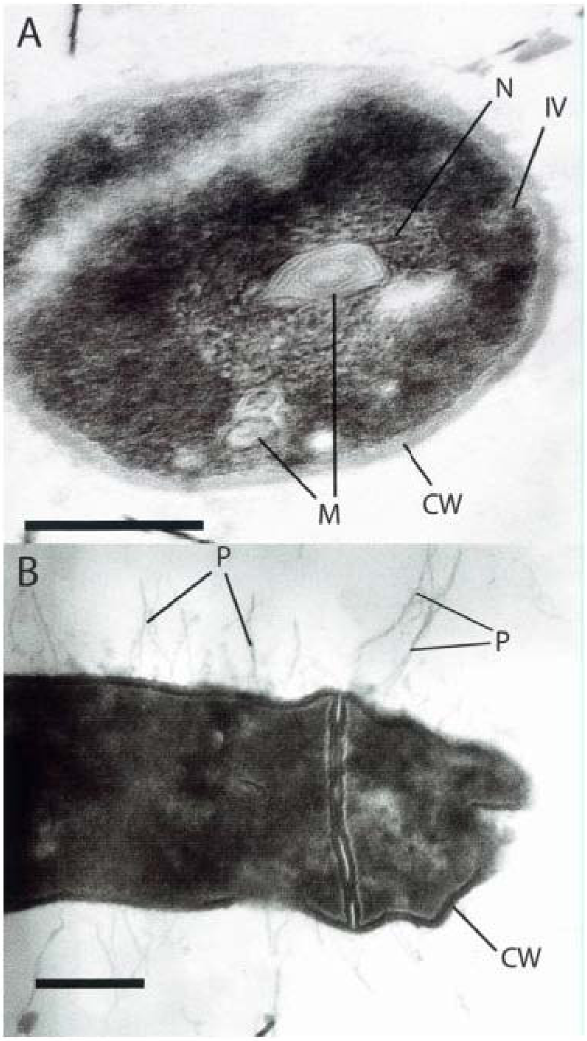

**FIGURE 9.** Thin-sectioning TEM showing a cell of mutant M5b (A) and M2b (B) after staining with $OsO_4$. Extracellular polymer (P) is absent in M5b, whereas it appears very weakly expressed on the cell surface of M2b. The cell wall (CW) of M2b is perforated or noncontinuous, and cell division has occurred near the polar position producing a smaller cell that appears devoid of chromosomal material. Membrane-bound invaginations (IV) and mesosome-like structures (M) appear more prominent in M5b. The largest of these is shown located within the nucleoid (N). Bar = 0.25 µm.

### DISCUSSION

In this study, we successfully isolated a number of mutant strains from *Rhodococcus* sp. 33 that exhibited reduced tolerance limits to benzene. A comparative evaluation of their characteristics with the *w-t*, including the observed differences in their cellular and colony morphologies, revealed interesting insights into the mechanisms that this *Rhodococcus* employs in tolerating benzene.

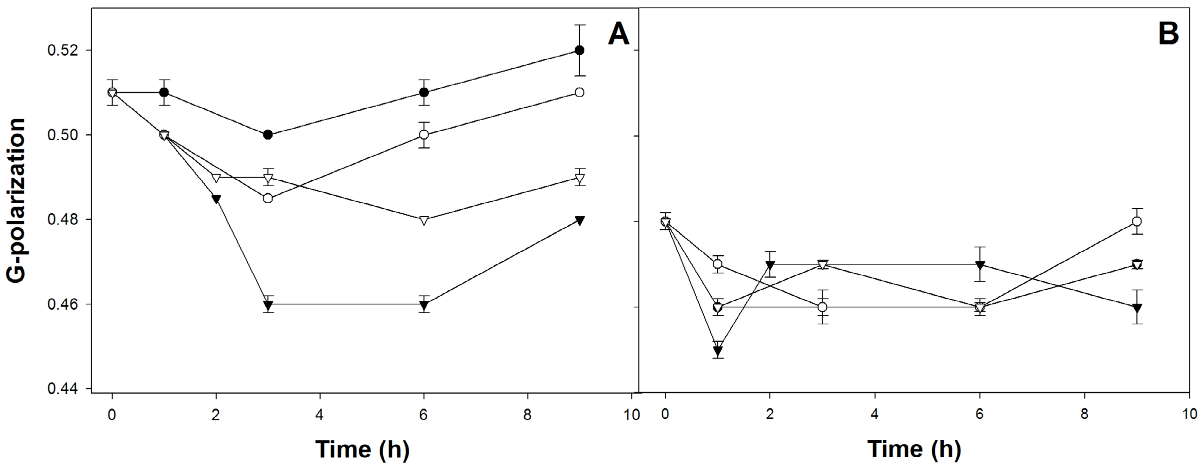

**FIGURE 10.** The change in GP of laurdan-labeled cells that were grown in the absence (nonadapted, A) or presence (adapted, B) of benzene, and then challenged with increasing concentrations of benzene — 300 mg/l (●), 600 mg/l (○), 1000 mg/l (▼), 1400 mg/l (▽). Where SD error bars are not shown, it is because they were smaller than the symbol.

Nonadapted cells of the *w-t* were found to tolerate shock concentrations of up to 1000 mg/l of presolubilized benzene, a concentration that would kill most other types of microorganisms[17]. Since the benzene-catabolizing enzymes of this organism are known to be suppressed during growth without benzene[12], the benzene-breakdown pathway is therefore unlikely to participate in tolerance. Furthermore, M1 and M3 were the only mutants to have lost the ability to degrade benzene as a sole carbon source, although they were still able to survive, and thus tolerate relatively high concentrations of benzene, 500 and 800 mg/l, respectively, over the duration of the experiment (8 h). Similarly, mutant strains of other *Rhodococcus* sp. have been isolated, displaying a defective ability to degrade benzene, although still found capable of tolerating high concentrations of this solvent[9], thus suggesting that degradation of organic solvents is not essential for tolerance. In the case of *Rhodococcus* sp. 33, mechanisms other than the benzene-catabolizing pathway must play important roles in conferring this organism with its high tolerance to benzene.

Previously, we reported that benzene induces an increase in the saturated:unsaturated ratio of fatty acids[18] and subsequent decrease in cell membrane fluidity[19] in this *Rhodococcus*. Such changes are known to mitigate the detrimental effects of solvents like benzene on the cell membranes of microorganisms[4]. This would explain why growing cultures of benzene-adapted cells were less affected when challenged with "shock" concentrations of >0.25% (v/v) benzene compared to nonadapted cells, which suffered marked changes in their growth rates. Monitoring cell membrane fluidity showed that membranes of adapted cells were in an enhanced physiological state that permitted them to tolerate higher concentrations of benzene. The initial drop in the GP values immediately after exposure, which corresponded to an increase in fluidity, can be attributed to benzene's tendency to partition within the cells' lipid bilayer[20]. The subsequent increase in the GP values can be interpreted as the cells' adaptive response, which may be to lower benzene's partitioning and ability to cause damage to the cell membrane or to instigate measures to counter the benzene-induced fluidization. As shown previously with *Rhodococcus* sp. 33[18], Ramos et al.[2] also measured a rapid increase in *trans* unsaturated fatty acids on exposure of *Pseudomonas putida* strain DOT-T1 to toluene, a mechanism that solvent-tolerant bacteria use to reduce the fluidity of their cell membranes.

Morphological studies revealed the *w-t* to produce a thick layer of polymer around the cells. In ruthenium red–stained cells, the high contrast observed by the polymer exhibits it to be a type of acidic polysaccharide, as confirmed previously by structural characterization of the polymer[21]. Polyanionic biopolymers can have useful commercial applications, such as in the sequestration of cationic dyes and heavy metals from contaminated effluents and wastewaters[22,23,24]. Previous work on the isolated polymer by Aizawa et al.[25] showed it to exhibit surfactant properties, similarly to polymers isolated from other rhodococci[26,27,28,29], and that addition of the polymer to rough mutant strains (i.e., lacking in their production of the polymer) increased both their survival and growth in media containing benzene. Interestingly, the pyruvic acid moiety of the polymer was suggested as a key factor in tolerance[25]. Collectively, since the mutant strains generated in the present study were found to have either partially or completely lost their ability to express cell surface polymer, their susceptibilities to benzene appear to be contributed by their lack of polymer expression. The polymer could possibly act as a sink for benzene accumulation, thus offering partial protection to other cellular structures. Exopolymers produced by marine microalgae, for example, have been shown to sequester lipophilic compounds, like polyaromatic hydrocarbons, from the surrounding seawater environment[30,31]. The polymer of *Rhodococcus* sp. 33 may play a similar role by sequestering molecules of benzene within its immediate environment, thereby reducing benzene contact with its cell membrane and conferring it with some degree of tolerance.

The origin of the many invaginations and mesosome-like structures in all of the mutants cannot be established at this time. Although these structures were also observed in some cells of the *w-t* (not shown), their numbers were much more abundant in the mutants. Mutants of common Gram-negative bacteria, such as *Escherichia coli*, have been shown to accumulate large numbers of intracellular mesosomes[32]. Although it is unknown whether these structures are a direct result of the mutation, or artifacts of fixation[33,34], they are generally more prominent in Gram-positive bacteria[35]. The finding of these structures inside cells of the isolated mutants suggests the existence of an abnormal cell membrane that, compared to that of the *w-t*, may be more susceptible to chemical solvents.

The formation of membrane vesicles during challenge of the *w-t* and mutants to various concentrations of benzene implies that the cell wall may have been a target of benzene. These vesicles, or "blebs", have been described as extensions of the cell membrane that protrude through openings in bacterial cell walls. By this mechanism, lipophilic solvents, such as toluene, have been used as a means to increase the permeability of bacterial cell walls for generating spheroplasts and whole cell lysates[36,37,38]. The cell wall of Gram-positive bacteria is quite a porous structure with a considerable tensile strength[39]. It is recognized to have a high molecular weight exclusion limit[40], enough to allow the passive diffusion of benzene into the cell. From our observations showing "bleb" formation during benzene exposure, it is apparent that benzene may directly interact with the murein layer and dissolve it. Since the viability of these cells was found to be considerably reduced, benzene-induced degradation of wall structure appears to be one mechanism involved in cell damage. Some of the mutants, notably M2b, M2c, M3, M5b, and M6b, also demonstrated characteristics to suggest that they suffered from a defective cell wall, and which may have contributed to their sensitivities to benzene. Such characteristics include their incomplete staining by the Gram reaction, as well as the pleomorphic appearance and striated transparency of these mutants under the phase contrast microscope. Indeed, pleomorphisms in bacteria have been associated with a loss in cell wall structure[41], and cell transparency can be compared to protoplast formation in the *w-t* during its treatment with the murein-active enzyme lysozyme.

Further to these observations on potential roles of the extracellular polymer and cell wall, one needs to consider the cell plasma membrane, the first dynamic structure to encounter extracellular benzene. As noted above, benzene tends to partition into the hydrophobic core of the cell membrane. Here we have shown that concentrations of benzene greater than 600 mg/l have a marked effect on membranes of the *Rhoodococcus w-t* strain grown in the absence of benzene. It can be seen that compensatory mechanisms are induced as the membrane fluidity starts to trend towards normal levels after several hours. Pre-exposure to benzene allows cells to adapt such that higher levels of benzene challenge have little effect on the cell membranes. Thus, the protective mechanisms partially elucidated here include extracellular polymer, cell wall, and plasma membrane.

The present study provides some valuable insight into the inherent and adaptive physiological mechanisms that confer *Rhodococcus* sp. 33 with its high tolerance to benzene. Future work to investigate the molecular basis underlying these mechanisms should initiate new opportunities to exploit the vast potential of this organism, for example, to enhance its metabolic capability for degrading other aromatic pollutants. Such a product would find important applications in the bioremediation of pollutants that often persist at highly contaminated sites.

### ACKNOWLEDGMENTS

We thank Prof. Mel Dixon (now retired) of the UNSW Electron Microscopy Unit for his valuable assistance, and Dr. Andrew Davies for assistance with the preparation of the phase and TEM images. This work was supported by a Special School of Microbiology and Immunology Research grant at the UNSW.
